# Analysis of Upper Facial Weakness in Central Facial Palsy Following Acute Ischemic Stroke

**DOI:** 10.3390/neurolint17010012

**Published:** 2025-01-19

**Authors:** Monton Wongwandee, Kantham Hongdusit

**Affiliations:** Department of Medicine, Faculty of Medicine, Srinakharinwirot University, Nakhon Nayok 26120, Thailand; h.kantham@gmail.com

**Keywords:** central facial palsy, acute ischemic stroke, Sunnybrook Facial Grading System

## Abstract

Background: Central facial palsy (CFP), resulting from upper motor neuron lesions in the corticofacial pathway, is traditionally characterized by the sparing of the upper facial muscles. However, reports of upper facial weakness in CFP due to acute ischemic stroke have challenged this long-held assumption. This study aimed to determine the prevalence of upper facial weakness in CFP and identify its associated clinical factors. Methods: In this cross-sectional study, we evaluated consecutive patients with acute ischemic stroke admitted to a university hospital in Thailand from January 2022 to June 2023. Full-face video recordings were analyzed using the Sunnybrook Facial Grading System. Upper facial weakness was defined as asymmetry in at least one upper facial expression. Multivariable logistic regression was performed to identify factors associated with upper facial weakness. Results: Of 108 patients with acute ischemic stroke, 92 had CFP, and among these, 70 (76%) demonstrated upper facial weakness. Tight eye closure (force and wrinkle formation, both 42%) was the most sensitive indicator for detecting upper facial weakness. Greater stroke severity, as reflected by higher NIHSS scores (adjusted odds ratio [aOR], 1.42; 95% CI 1.07–1.88) and the presence of lower facial weakness (aOR, 6.56; 95% CI 1.85–23.29) were significantly associated with upper facial involvement. Although upper facial weakness was generally milder than lower facial weakness, its severity correlated with increasing lower facial asymmetry during movement. Conclusions: Contrary to traditional teaching, upper facial weakness is common in CFP due to acute ischemic stroke. The severity of stroke and the presence of lower facial weakness are key predictors of upper facial involvement. These findings underscore the need for clinicians to reconsider the diagnostic paradigm, recognizing that upper facial weakness can occur in CFP. Enhanced awareness may improve diagnostic accuracy, inform treatment decisions, and ultimately lead to better patient outcomes.

## 1. Introduction

Facial palsy is clinically divided into two types: central (upper motor neurons) and peripheral (lower motor neurons). Central facial palsy (CFP) results from lesions in the corticofacial pathway (e.g., ischemic stroke), whereas peripheral facial palsy (PFP) involves lesions in the facial nucleus or the seventh cranial nerve (e.g., Bell’s palsy). Differentiating between CFP and PFP in patients presenting with facial palsy is a critical initial step in achieving an accurate diagnosis. Traditionally, CFP has been understood to manifest as weakness in the lower half of the face contralateral to the lesion (mouth), while PFP typically results in complete ipsilateral facial weakness involving the upper and lower face (forehead, eye closure, mouth). This classic teaching reflects the concept that the upper face is spared in CFP due to its bilateral cortical innervation [1]. However, clinical observations, including some studies and case reports, suggest that upper facial weakness can occur in patients with CFP due to stroke [2,3,4,5,6]. The proportion of patients showing upper facial weakness varies across studies, likely due to differences in participant characteristics and methods of facial assessment. This study aims to systematically determine the prevalence of upper facial weakness using standardized facial function measurements and to identify factors associated with this condition in patients with CFP due to acute ischemic stroke. The findings could aid clinicians in better managing patients with facial palsy and contribute to a deeper understanding of the mechanisms underlying CFP.

## 2. Materials and Methods

### 2.1. Study Design and Setting

We conducted an observational, cross-sectional study in a single-center, tertiary-care university hospital in Thailand. From 1 January 2022 to 30 June 2023, all patients aged 18 years or older, diagnosed with acute ischemic stroke, and admitted to the stroke unit of the H.R.H. Princess Maha Chakri Sirindhorn Medical Center were prospectively and consecutively recruited into the study. Eligibility was assessed. Patients with a history of prior facial palsy or stroke, peripheral facial palsy due to lower pontine infarction, those who were intubated, or those unable to follow commands for facial expressions (e.g., non-cooperative, aphasic, or with altered mental status) were excluded.

### 2.2. Facial Muscle Function Assessment

The Sunnybrook Facial Grading System was used to quantitatively assess facial muscle functions (i.e., rating scales), specifically focusing on two components: (1) Resting Symmetry and (2) Symmetry of Voluntary Movement [7].

1. Resting Symmetry: This component evaluates the symmetry of three facial features at rest: the palpebral fissure, nasolabial fold, and mouth corner.
2. Symmetry of Voluntary Movement: Five standard facial expressions are assessed: forehead wrinkles, gentle eye closure, open-mouth smile, snarl, and lip pucker.

Additionally, we included an assessment of tight eye closure, examining three features: the symmetry of wrinkles, buried eyelashes, and resistance against force.

Facial asymmetry in any expression indicates muscle weakness, with greater asymmetry corresponding to more severe weakness. A higher score in the Resting Symmetry part but a lower score in the movement part signifies greater severity of facial weakness. Upper facial weakness was determined by asymmetry in the palpebral fissure at rest, or the forehead wrinkles or eye closures during movement. Lower facial weakness was identified by asymmetry in the nasolabial fold or mouth corner at rest, or smile, snarl, or lip pucker during movement. Full-face video recordings were made during admission using an iPhone SE (2nd generation) camera for later assessment, ensuring accurate evaluation.

### 2.3. Stroke Lesion Localization

The Alberta Stroke Program Early CT Score (ASPECTS) was used to systematically localize ischemic stroke lesions in brain imaging. The original ASPECTS system evaluates 10 specific regions within the middle cerebral artery (MCA) territory, which include the MCA cortex (M1–M6), caudate, lentiform nucleus, internal capsule, and insula ribbon [8]. To improve localization precision, we expanded our assessment to include regions in the anterior cerebral artery (ACA) territory and posterior circulation, using the posterior circulation Acute Stroke Prognosis Early CT Score (pc-ASPECTS), which includes the thalamus, posterior cerebral artery (PCA) territory, midbrain, pons, and cerebellum [9].

To prevent bias from patient clinical data influencing stroke lesion localization or facial muscle function assessment, both evaluations were conducted only after all patients were recruited. Lesion localization on the Picture Archiving and Communication System (PACS) was completed for all patients first, followed by the assessment of facial muscle function via video recordings [10]. A neurologist with over 10 years of experience independently evaluated the brain images, while facial function was assessed by a neurologist and an internist, with disagreements resolved by consensus.

### 2.4. Clinical Data

Demographic data, stroke risk factors, prestroke-modified Rankin Scale (mRS)—an ordinal functional disability scale with scores ranging from 0 (no symptoms) to 6 (death)—as well as the date and time of stroke onset were collected through patient interviews and medical records [11]. The National Institutes of Health Stroke Scale (NIHSS), which ranges from 0 to 42, with higher scores indicating more severe neurologic deficits, was assessed on the same day as the video recording of facial muscle functions [12].

### 2.5. Statistical Analysis

We reported the prevalence of upper or lower facial weakness as absolute numbers and percentages of total patients with facial weakness. Differences in facial symmetry scores were evaluated using the Friedman test, with post hoc analysis performed using Wilcoxon signed-rank tests. Spearman’s rank correlation was used to analyze relationships between the severity of facial weakness in different expressions. Exploratory univariable analysis was conducted to identify associations between various variables and upper facial weakness. Categorical data were analyzed using the Chi-square test. For continuous data, Student’s *t*-test was applied when the data followed a normal distribution, and the Mann–Whitney U test was used when the data were not normally distributed. Multivariable logistic regression analysis was performed to predict upper facial weakness, including candidate variables based on prior knowledge and those with *p* < 0.1 from the exploratory univariable analysis. A stepwise backward likelihood-ratio method was used for variable selection, and the logistic regression was repeated with interaction terms among potential predictors. Multicollinearity was assessed using multiple linear regression analysis to identify highly correlated factors. The final model was validated by assessing calibration with the Hosmer–Lemeshow test and evaluating discriminative ability through the area under the receiver operating characteristic (ROC) curve, thereby confirming its fit to the data. No assumptions were made regarding missing data; thus, a complete case analysis was performed. Statistical analyses were conducted using IBM SPSS Statistics for Windows, Version 25.0.

## 3. Results

### 3.1. Patient Characteristics

**Figure 1:**
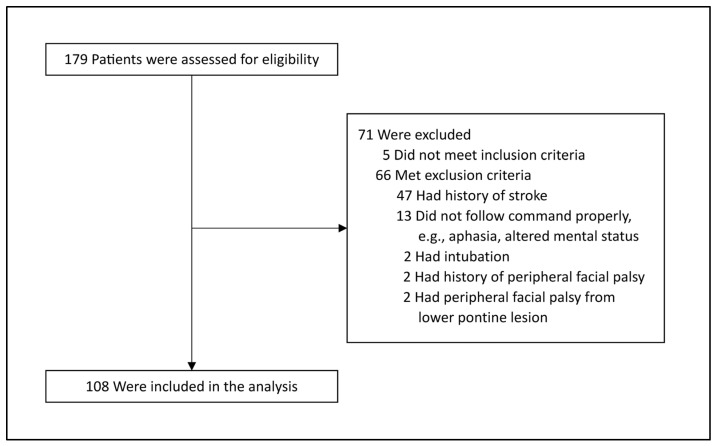
Enrollment and inclusion. All patients aged 18 years or older, diagnosed with acute ischemic stroke, and admitted to the stroke unit were prospectively and consecutively recruited into the study.

A total of 179 patients with acute ischemic stroke were assessed for eligibility. Seventy-one participants were excluded based on the defined criteria, resulting in a final analysis of 108 participants (Figure 1). The mean age of the participants was 63 years, with 51% being male. Hypertension was identified as the most common risk factor for stroke (78%). The median mRS score was 0, and the median NIHSS score was 2. CT scans were the predominant imaging modality (89%). Among the ASPECTS regions, the M5 region was the most frequently identified lesion location (44%). Detailed patient characteristics are presented in Table 1.

### 3.2. Prevalence and Severity of Upper Facial Weakness

Facial weakness was observed in 92 patients (85%). Of these, 70 patients (76%) had upper facial weakness, while 88 (96%) had lower facial weakness. Sixty-six patients (72%) exhibited both upper and lower facial weakness, with four (4%) displaying isolated upper facial weakness and twenty-two (24%) exhibiting isolated lower facial weakness. Tight eye closure force and wrinkles were the most sensitive indicators of upper facial weakness (both 42%). Detailed prevalence and severity of facial weakness across different facial muscle functions are provided in Table 2.

**Figure 2:**
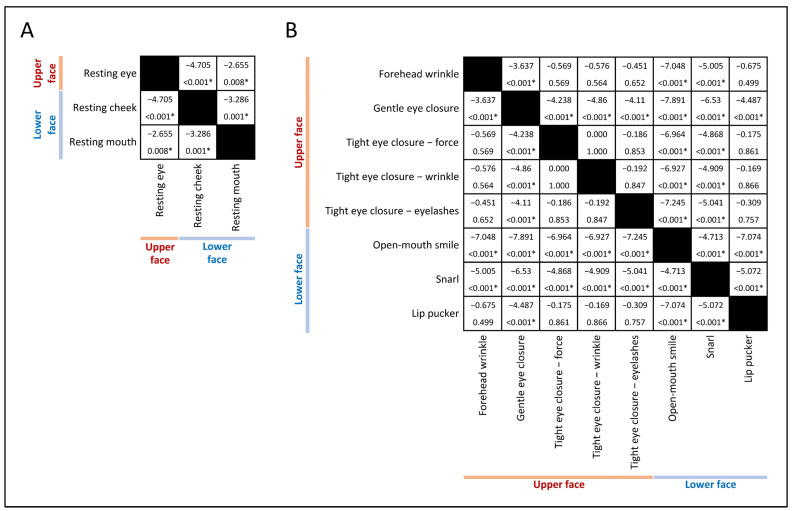
Comparative analysis of facial muscle function at rest and during movement. The matrices depict pairwise comparisons of facial symmetry scores between various facial functions, both at rest (**A**) and during movement (**B**) across upper and lower face regions. Each cell in the matrix displays the z-score (top value) and the corresponding *p*-value (bottom value) derived from Wilcoxon signed-rank tests with Bonferroni correction. Statistically significant differences are denoted with an asterisk (*), applying a threshold of *p* < 0.017 for rest and *p* < 0.002 for movement. The threshold is calculated by 0.05 divided by the number of pairwise comparisons. Black cells: ‘Not applicable’ for self-comparisons.

**Figure 3:**
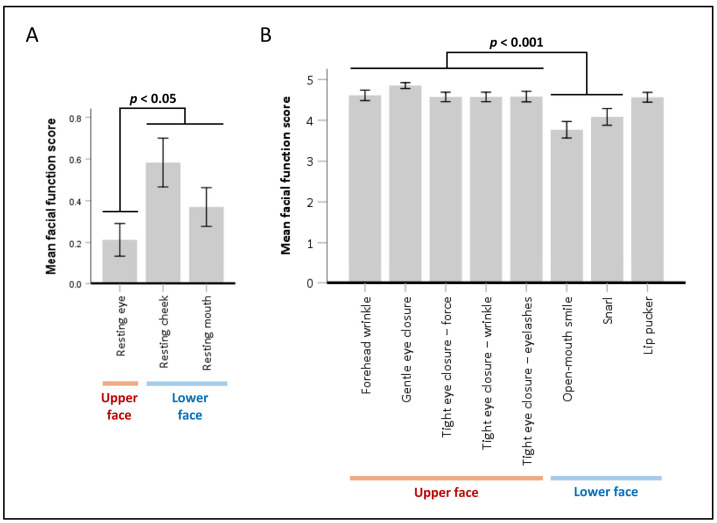
Mean facial function scores at rest and during movement. The bar graphs display mean facial function scores, representing facial symmetry, at rest (**A**) and during movement (**B**) across upper and lower face regions. Higher scores at rest and lower scores during movement indicate greater severity of facial asymmetry. Error bars represent 95% confidence intervals. Statistically significant differences in pairwise scores between upper and lower facial functions, along with corresponding *p*-values, were determined using Wilcoxon signed-rank tests with Bonferroni correction.

Significant differences in facial symmetry scores were found across various facial muscle functions, both at rest (χ^2^(2) = 27.522, *p* < 0.001) and during movement (χ^2^(7) = 220.608, *p* < 0.001). Post hoc analysis using Wilcoxon signed-rank tests with Bonferroni correction resulted in a significance level set at *p* < 0.017 at rest and *p* < 0.002 during movement (Figure 2). Generally, upper facial muscle weakness was less severe than lower facial muscle weakness (Figure 3). At rest, asymmetry in the palpebral fissure was significantly less severe than in the nasolabial fold and mouth corner. During movement, asymmetry in forehead wrinkles, gentle eye closure, and tight eye closure were significantly less severe than in an open-mouth smile and snarl, but not significantly different from lip pucker.

**Figure 4:**
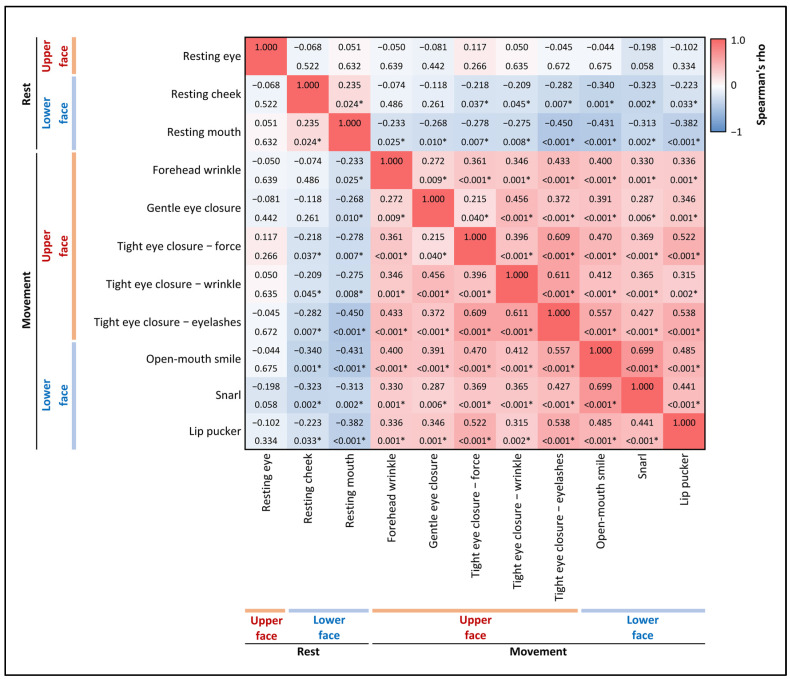
Correlation analysis of facial muscle functions at rest and during movement. This heatmap presents the correlations between various facial muscle functions, both at rest and during movement, across the upper and lower face regions. Each cell in the matrix displays Spearman’s rho (top value) and the corresponding *p*-value (bottom value). The color gradient represents the strength and direction of the correlations, with blue indicating negative correlations and red indicating positive correlations. Statistically significant correlations (*p* < 0.05) are marked with an asterisk (*).

Spearman’s rank correlation analysis assessed relationships among various facial muscle function scores at rest and during movement across upper and lower face regions. Higher facial symmetry scores in the upper face were significantly correlated with higher scores in the lower face during movement but not at rest (Figure 4).

### 3.3. Factors Associated with Upper Facial Weakness

**Figure 5:**
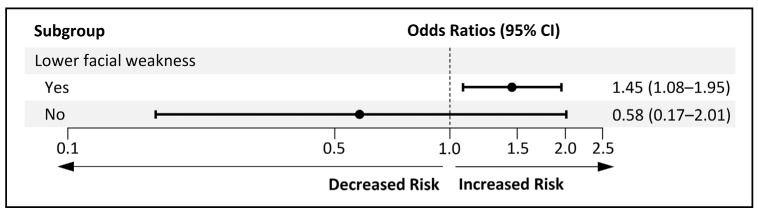
Subgroup analysis of upper facial weakness in patients with central facial palsy from acute ischemic stroke. The figure shows a subgroup analysis of patients with central facial palsy due to acute ischemic stroke, stratified by lower facial weakness. Odds ratios (OR) and 95% confidence intervals (CI) reflect the likelihood of upper facial weakness for each subgroup, per one-point increase in National Institutes of Health Stroke Scale (NIHSS) score.

Univariable analysis results are presented in Table 1. Six variables with *p* < 0.1—NIHSS, presence of lower facial weakness, M5 lesion (from ASPECTS), lentiform nucleus lesion, cerebellar lesion, and the presence of dilated cardiomyopathy—were selected as candidates for the multivariable logistic regression model. Additionally, ACA lesion and lesion side were included as candidate variables based on prior knowledge. No collinearity was found between variables (variance inflation factor [VIF] < 10, tolerance > 0.1) (Table S1). Factors associated with upper facial weakness included the NIHSS score (adjusted odds ratio [aOR] 1.42; 95% confidence interval [CI] 1.07−1.88) and the presence of lower facial weakness (aOR 6.56; 95% CI 1.85−23.29) (Table 3). The Hosmer–Lemeshow test indicated good model fit (χ^2^ = 10.738, *p* = 0.097). The area under the receiver-operating characteristic curve (AUROC) was 0.78 (95% CI 0.69–0.87), with a sensitivity of 94.3%, specificity of 42.1%, and accuracy of 75.9% (Figure S1). Logistic regression was repeated with an interaction term between NIHSS and the presence of lower facial weakness, revealing a significant interaction (OR 3.53, 95% CI 1.45–8.59). In patients with lower facial weakness, a higher NIHSS score increased the likelihood of upper facial weakness (OR 1.45, 95% CI 1.08–1.95). However, in patients without lower facial weakness, the NIHSS score was not associated with upper facial weakness (OR 0.58, 95% CI, 0.17–2.01) (Figure 5).

## 4. Discussion

### 4.1. Key Findings

This study reveals that upper facial weakness is common in patients with CFP due to acute ischemic stroke. Among those with CFP, 76% exhibit upper facial weakness, as indicated by asymmetrical facial expressions, although this weakness is notably less severe than that of the lower face. The most sensitive indicators of upper facial weakness were tight eyelid closure force and wrinkles (42% for both), while the resting palpebral fissure was the least sensitive (16%). The NIHSS score and the presence of lower face weakness were significantly associated with upper facial weakness (aOR = 1.42 and 6.56, respectively), but the location of the stroke lesion was not related. However, subgroup analysis showed that a higher NIHSS score increased the risk of upper facial weakness only among those with lower facial weakness. Additionally, we observed a correlation between the severity of upper and lower facial weaknesses.

### 4.2. Comparison with Previous Studies

Previous case reports have described upper facial involvement in CFP due to stroke [3,4]. However, few studies have systematically assessed the prevalence of upper facial weakness, with reported rates ranging from 7% to 54% in patients with CFP due to acute ischemic stroke—lower than those in our study [2,5,6]. The differences likely arise from variations in assessment methods and criteria for defining weakness. For example, one study graded the force of forehead elevation and eyelid closure against manual resistance on a scale from 0 to 4, with any score of 1 or higher indicating weakness [5]. Another study used an ergometer to measure eye closure strength, defining weakness as a bilateral strength difference exceeding 0.05 kg of force sustained for over three days [2]. Another study clinically examined movements of forehead and eye closure but did not define the weakness [6]. Our methodology likely yielded a higher prevalence of upper facial weakness due to the sensitivity of our assessment approach. We applied the Sunnybrook Facial Grading System to evaluate six upper facial expressions, including the resting palpebral fissure and movements of the forehead, gentle eye closure, and tight eye closure (force, wrinkles, and buried eyelashes). This system is more practical for clinical settings than prior methods and has shown reliability in assessing CFP in subacute stroke patients [13]. Variations in patient characteristics among studies may also contribute to differing prevalence rates. Some studies included only ischemic stroke patients with cortical lesions, while others included both ischemic and hemorrhagic strokes with lesion locations spanning the supratentorial cortical and subcortical regions [2,5,6]. Our study expanded these criteria to include ischemic strokes with supra- and infratentorial, cortical, and subcortical lesions. However, our participants generally had less severe strokes compared to those in previous studies [2]. The explanation why doctors often do not recognize the upper facial weakness in CFP in stroke patients despite its prevalence was probable that the upper facial weakness was mild and apparently less severe than the lower face weakness.

### 4.3. Facial Motor Control Pathways and Correlated Findings

Traditionally, it was believed that the ventral lateral primary motor cortex (M1) was the sole contributor to corticofacial projections, with bilateral M1 innervation to the upper facial nucleus allowing the intact hemisphere to compensate, and contralateral-only innervation to the lower facial nucleus causing classic “central” lower facial weakness in MCA infarctions [1,14]. However, recent studies of surgical resection, neuroanatomy, and neuroimaging show a multifocal cortical representation involving M1, the ventral lateral premotor cortex (LPMCv), the supplementary motor cortex (M2), and the rostral (M3) and caudal (M4) cingulate motor cortices, all of which project to the four subnuclei of the facial nucleus (medial, lateral, dorsal, and intermediate) with variable bilateral input [15,16,17,18]. In particular, M3 bilaterally innervates the dorsal and intermediate subnuclei responsible for upper facial movement; its fibers travel rostrally in the corona radiata, descend through the anterior limb and genu of the internal capsule, and align medially in the crus cerebri [19].

Our study demonstrated a strong association between upper facial weakness and the presence of lower facial weakness, as well as a correlation between their severities. Additionally, upper facial weakness was associated with stroke severity (NIHSS) in patients with lower facial weakness. These findings suggest that while the cortical representations and corticofacial pathways of the upper and lower facial muscles are not in the same regions, they do partly overlap. In humans, the lateral motor areas (M1 and LPMCv) are known as cortical representations for lower facial muscles, indicating that upper facial muscle representations likely lie outside these areas. This concept is supported by animal studies showing that the cortical representation of upper facial muscles resides in the medial motor area (M3) [18]. Furthermore, a human clinico–radiological study showed that ACA-territory medial brain infarction was associated with eyelid closure weakness [5]. However, many stroke patients with MCA-territory infarcts also exhibit upper facial weakness. This observation may be explained by findings that the corticofacial pathway for upper facial muscles, while originating from the ACA-territorial M3 area, also passes through MCA-territorial brain regions, such as the anterior limb and genu of the internal capsule [19].

### 4.4. Limitations

This present study has several limitations: (1) Normal variations in minimal facial asymmetry may be misinterpreted as facial palsy due to the sensitivity of the grading system. (2) Stroke lesions were primarily assessed by CT, which is less sensitive than MRI. (3) Most lesion locations were determined using the ASPECTS system, which relies on only two axial imaging cuts, potentially missing lesions outside these views. (4) Only new cerebral infarctions were included in the analysis, while silent, old ischemic lesions could have confounded the findings on facial weakness. (5) We excluded patients who were unable to follow commands, such as those with altered consciousness or aphasia, which are more common in severe stroke, limiting the generalizability of the findings to real clinical settings. (6) Only two patients with ACA-territory strokes were included, limiting the power to analyze associations with upper facial weakness.

### 4.5. Clinical Implications

Our study’s results are valuable for clinicians treating stroke patients, as upper facial weakness is commonly found in patients with CFP from acute ischemic stroke. It is especially evident when lower facial muscles are severely weak or neurological deficits are extensive. The presence of both upper and lower facial weakness in patients with CFP may lead doctors, who adhere to traditional diagnostic guidelines, to misinterpret it as peripheral facial palsy. Notably, upper facial muscle weakness in CFP is typically less severe than lower facial weakness, and ipsilateral upper extremity weakness may also be present. To improve study robustness in assessing associated factors, especially lesion locations, future research should prioritize MRI-based lesion mapping, accounting for both new and old lesions rather than solely relying on the ASPECTS system. Moreover, future studies should aim to recruit ischemic stroke patients from a broader range of arterial territories, particularly the ACA territory.

## 5. Conclusions

Upper facial weakness is common in patients with CFP due to acute ischemic stroke, although it is typically less severe than lower facial weakness. The NIHSS score and the presence of lower facial weakness were significant factors associated with upper facial weakness. The increased severity of upper facial weakness correlated with greater severity of lower facial weakness during movement. Clinicians should consider upper facial weakness in patients with CFP from acute ischemic stroke, particularly when stroke severity or lower facial weakness is marked.

## Figures and Tables

**Table 1:** Patient characteristics according to the presence of upper facial weakness *.

Characteristic	Total (*N* = 108)	Upper Facial Weakness (*N* = 70)	No Upper Facial Weakness (*N* = 38)	Measure of Effect †	Unadjusted Effect Size (95% CI) †	*p*-Value
Age—yr	63.1 ± 11.8	63.7 ± 11.5	62.1 ± 12.5	Mean difference	1.59 (−6.33–3.15)	0.507
Male sex—no. (%)	55 (50.9)	38 (54.3)	17 (44.7)	Odds ratio	1.47 (0.66–3.24)	0.343
Median prestroke mRS (IQR) ‡	0.0 (0.0–0.0)	0.0 (0.0–0.0)	0.0 (0.0–0.0)	*r*	−0.08	0.432
Median NIHSS score on the day of facial recording (IQR) §	2.0 (1.0−4.0)	3.0 (1.0–5.0)	2.0 (1.0–3.0)	*r*	−0.33	0.001
Risk factor for ischemic stroke—no. (%)						
Hypertension	86 (79.6)	55 (78.6)	31 (81.6)	Odds ratio	0.83 (0.31–2.25)	0.711
Dyslipidemia	67 (62.0)	43 (61.4)	24 (63.2)	Odds ratio	0.93 (0.41–2.10)	0.860
Diabetes mellitus	45 (41.7)	32 (45.7)	13 (34.2)	Odds ratio	1.62 (0.71–3.67)	0.247
Smoking	34 (31.5)	23 (32.9)	11 (28.9)	Odds ratio	1.20 (0.51–2.84)	0.676
Chronic kidney disease	14 (13.0)	8 (11.4)	6 (15.8)	Odds ratio	0.69 (0.22–2.15)	0.519
Atrial fibrillation	8 (7.4)	5 (7.1)	3 (7.9)	Odds ratio	0.90 (0.20–3.98)	0.887
Coronary artery disease	7 (6.5)	3 (4.3)	4 (10.5)	Odds ratio	0.38 (0.08–1.80)	0.208
Dilated cardiomyopathy	2 (1.9)	0 (0.0)	2 (5.3)	NA	NA	0.053
Rheumatic heart disease	2 (1.9)	2 (2.9)	0 (0.0)	NA	NA	0.293
Left ventricular thrombus	1 (0.9)	0 (0.0)	1 (2.6)	NA	NA	0.173
Intravenous thrombolysis—no. (%)	15 (13.9)	10 (14.3)	5 (13.2)	Odds ratio	1.10 (0.35–3.49)	0.871
Imaging for localization—no. (%)						
CT	96 (88.9)	63 (90.0)	33 (86.8)	Odds ratio	1.36 (0.40–4.63)	0.618
MRI	12 (11.1)	7 (10.0)	5 (13.2)			
Side of lesions—no. (%) ¶						
Right	32 (33.3)	23 (37.1)	9 (26.5)	Odds ratio	1.64 (0.65–4.11)	0.291
Left	64 (66.7)	39 (62.9)	25 (73.5)			
Localization by tentorium cerebelli—no. (%) ¶						
Supratentorium	84 (87.5)	56 (90.3)	28 (82.4)	Odds ratio	2.00 (0.59–6.77)	0.259
Infratentorium	12 (12.5)	6 (9.7)	6 (17.6)			
Localization by cerebral cortex						
Cortical areas	17 (20.2)	9 (16.1)	8 (28.6)	Odds ratio	0.48 (0.16–1.42)	0.179
Subcortical areas	67 (79.8)	47 (83.9)	20 (71.4)			
Localization by the ASPECTS system—no. (%) ¶ǁ						
M1	7 (6.5)	6 (8.6)	1 (2.6)	Odds ratio	3.47 (0.40–29.9)	0.231
M2	3 (2.8)	2 (2.9)	1 (2.6)	Odds ratio	1.09 (0.10–12.4)	0.946
M3	5 (4.6)	4 (5.7)	1 (2.6)	Odds ratio	2.24 (0.24–20.81)	0.467
M4	6 (5.6)	5 (7.1)	1 (2.6)	Odds ratio	2.85 (0.32–25.30)	0.328
M5	47 (43.5)	35 (50.0)	12 (31.6)	Odds ratio	2.17 (0.95–4.96)	0.065
M6	8 (7.4)	5 (7.1)	3 (7.9)	Odds ratio	0.90 (0.20–3.98)	0.887
I	12 (11.1)	7 (10.0)	5 (13.2)	Odds ratio	0.73 (0.22–2.49)	0.618
C	3 (2.8)	2 (2.9)	1 (1.6)	Odds ratio	1.09 (0.10–12.41)	0.946
L	14 (13.0)	12 (17.1)	2 (5.3)	Odds ratio	3.72 (0.79–17.61)	0.079
IC	27 (25.0)	20 (28.6)	7 (18.4)	Odds ratio	1.77 (0.67–4.68)	0.245
A	2 (1.9)	1 (1.4)	1 (2.6)	Odds ratio	0.54 (0.03–8.82)	0.658
P	4 (3.7)	2 (2.9)	2 (5.3)	Odds ratio	0.53 (0.07–3.92)	0.527
Thalamus	7 (6.5)	3 (4.3)	4 (10.5)	Odds ratio	0.38 (0.08–1.80)	0.208
Midbrain	1 (0.9)	0 (0.0)	1 (2.6)	NA	NA	0.173
Pons	8 (7.4)	7 (10.0)	1 (2.6)	Odds ratio	4.11 (0.49–34.74)	0.163
Cerebellum	8 (7.4)	2 (2.9)	6 (15.8)	Odds ratio	0.16 (0.03–0.82)	0.014
Median interval between stroke onset and facial assessment recording (IQR)—hour	62.5 (30.6−96.0)	61.5 (28.0–96.0)	64.8 (38.9–109.0)	*r*	−0.05	0.578
Lower face weakness—no. (%)	88 (81.5)	66 (94.3)	22 (57.9)	Odds ratio	12.0 (3.62–39.73)	<0.001

* Associations between patient characteristics and the presence of upper facial weakness were analyzed using Chi-square test for categorical data, and Student’s *t*-test or Mann–Whitney U test for continuous data depending on the data distribution. Plus–minus values are means ± SD. IQR denotes interquartile range, CI confidence interval, CT computed tomography, MRI magnetic resonance imaging, NA = not applicable. † For Mann–Whitney U test, the effect size *r* does not provide 95% CI. Odds ratio was not calculated if there were zero subjects in any patient group. ‡ Scores on the modified Rankin scale (mRS) of functional disability range from 0 (no symptoms) to 6 (death). § Scores on the National Institutes of Health Stroke Scale (NIHSS) range from 0 to 42, with higher scores indicating more severe neurologic deficits. ¶ Twelve patients had no brain lesions based on the CT brain. ǁ Alberta Stroke Program Early Computed Tomography Score (ASPECTS) system divides brain in middle cerebral artery (MCA) territory into ten regions (M1 = anterior MCA cortex, M2 = MCA cortex lateral to insular ribbon, M3 = posterior MCA cortex; M4, M5, and M6 = anterior, lateral, and posterior MCA territories immediately superior to M1, M2, M3, rostral to basal ganglia; I = insular ribbon, C = caudate, L = lentiform, IC = internal capsule). The anterior cerebral artery circulation (A) and the posterior circulation Acute Stroke Prognosis Early CT score (pc-ASPECTS) system including the thalamus, posterior cerebral artery circulation (P), midbrain, pons, and cerebellum were added.

**Table 2:** Facial muscle functions in patients with central facial palsy from acute ischemic stroke *.

Facial Function	Number (%) † (*N* = 92)	Median Score ‡ (IQR)
Resting Symmetry		
Palpebral fissure		0.0 (0.0−0.8)
Normal [0] §	69 (75.0)	
Abnormal	23 (25.0)	
Wide [1]	23 (25.0)	
Nasolabial fold		1.0 (0.0−1.0)
Normal [0]	36 (39.1)	
Abnormal	56 (60.9)	
Less pronounced [1]	49 (53.3)	
Absent [2]	7 (7.6)	
Mouth corner		0.0 (0.0−1.0)
Normal [0]	52 (56.5)	
Abnormal	40 (43.5)	
Corner dropped/pull up [1]	40 (43.5)	
Voluntary Movement Symmetry		
Forehead wrinkles		5.0 (4.0−5.0)
Normal symmetry [5] §	60 (65.2)	
Abnormal	32 (34.8)	
Mild asymmetry [4]	24 (26.1)	
Moderate symmetry [3]	6 (6.5)	
Severe asymmetry [2]	2 (2.2)	
Gross asymmetry [1]	0 (0.0)	
Gentle eye closure		5.0 (5.0−5.0)
Normal symmetry [5]	77 (83.7)	
Abnormal	15 (16.3)	
Mild asymmetry [4]	14 (15.2)	
Moderate symmetry [3]	1 (1.1)	
Severe asymmetry [2]	0 (0.0)	
Gross asymmetry [1]	0 (0.0)	
Tight eye closure—force		5.0 (4.0−5.0)
Normal symmetry [5]	53 (57.6)	
Abnormal	39 (42.4)	
Mild asymmetry [4]	32 (34.8)	
Moderate symmetry [3]	7 (7.6)	
Severe asymmetry [2]	0 (0.0)	
Gross asymmetry [1]	0 (0.0)	
Tight eye closure—wrinkles		5.0 (4.0−5.0)
Normal symmetry [5]	53 (57.6)	
Abnormal	39 (42.4)	
Mild asymmetry [4]	32 (34.8)	
Moderate symmetry [3]	7 (7.6)	
Severe asymmetry [2]	0 (0.0)	
Gross asymmetry [1]	0 (0.0)	
Tight eye closure—eyelashes		5.0 (4.0−5.0)
Normal symmetry [5]	58 (63.0)	
Abnormal	34 (37.0)	
Mild asymmetry [4]	24 (26.1)	
Moderate symmetry [3]	9 (9.8)	
Severe asymmetry [2]	1 (1.1)	
Gross asymmetry [1]	0 (0.0)	
Open mouth smile		4.0 (3.0−4.0)
Normal symmetry [5]	13 (14.1)	
Abnormal	79 (85.9)	
Mild asymmetry [4]	42 (45.7)	
Moderate symmetry [3]	24 (26.1)	
Severe asymmetry [2]	9 (9.8)	
Gross asymmetry [1]	4 (4.3)	
Snarl		4.0 (3.3−5.0)
Normal symmetry [5]	31 (33.7)	
Abnormal	61 (66.3)	
Mild asymmetry [4]	38 (41.3)	
Moderate symmetry [3]	12 (13.0)	
Severe asymmetry [2]	7 (7.6)	
Gross asymmetry [1]	4 (4.3)	
Lip pucker		5.0 (4.0−5.0)
Normal symmetry [5]	53 (57.6)	
Abnormal	39 (42.4)	
Mild asymmetry [4]	31 (33.7)	
Moderate symmetry [3]	8 (8.7)	
Severe asymmetry [2]	0 (0.0)	
Gross asymmetry [1]	0 (0.0)	

* The Sunnybrook Facial Grading System was applied to assess facial muscle functions by means of scoring (i.e., rating scales) for the symmetry of each facial function. Patients were identified as having facial palsy if there was asymmetry in at least one facial function. There were two parts of evaluation including the Resting Symmetry and the Voluntary Movement Symmetry. † Number of patients who had a particular score in each facial function. ‡ Median score of each facial function. § Number in parentheses [ ] is the score rated for each facial function. A higher score in the Resting Symmetry part and a lower score in the Voluntary Movement Symmetry part signifies greater severity of facial palsy. For the scores in the Voluntary Movement Symmetry part, 5 = Complete movement, 4 = Almost complete movement, 3 = Mild excursion, 2 = Slight movement, 1 = No movement.

**Table 3:** Adjusted odds ratios (ORs) for factors associated with upper facial weakness *.

Predictor Variable	Upper Facial Weakness (*N* = 70)	No Upper Facial Weakness (*N* = 38)	Adjusted OR (95%CI)	*p*-Value
Median NIHSS score (IQR)	3.0 (1.0–5.0)	2.0 (1.0–3.0)	1.42 (1.07−1.88) †	0.016
Lower face weakness—no. (%)	66 (94.3)	22 (57.9)	6.56 (1.85−23.29)	0.004

* This table shows adjusted odds ratios (ORs) from a multiple logistic regression analysis, evaluating the association between selected predictors and the likelihood of upper facial weakness in patients with central facial palsy due to acute ischemic stroke. Variables included in the model were selected using stepwise backward likelihood-ratio methodology. ORs are adjusted for all candidate variables in the model. NIHSS denotes National Institutes of Health Stroke Scale. † The odds ratio per one-point increase in NIHSS score is shown.

## Data Availability

Data are contained within the article or Supplementary Material.

## Supplementary Materials

The following supporting information can be downloaded at: https://www.mdpi.com/article/10.3390/neurolint17010012/s1, Figure S1: Receiver-operating characteristic (ROC) curve for predicting upper facial weakness in patients with central facial palsy due to acute ischemic stroke; Table S1: Multicollinearity analysis of candidate variables for multiple logistic regression.
