# Supplementary material for: Analysis of Upper Facial Weakness in Central Facial Palsy Following Acute Ischemic Stroke
Source: Neurol Int. 2025 Jan 19;17(1):12. doi: 10.3390/neurolint17010012 (PMC11767383; doi:10.3390/neurolint17010012)
Supplement: Supplementary file 1 [file neurolint-17-00012-s001.zip › Figure S1.pdf]

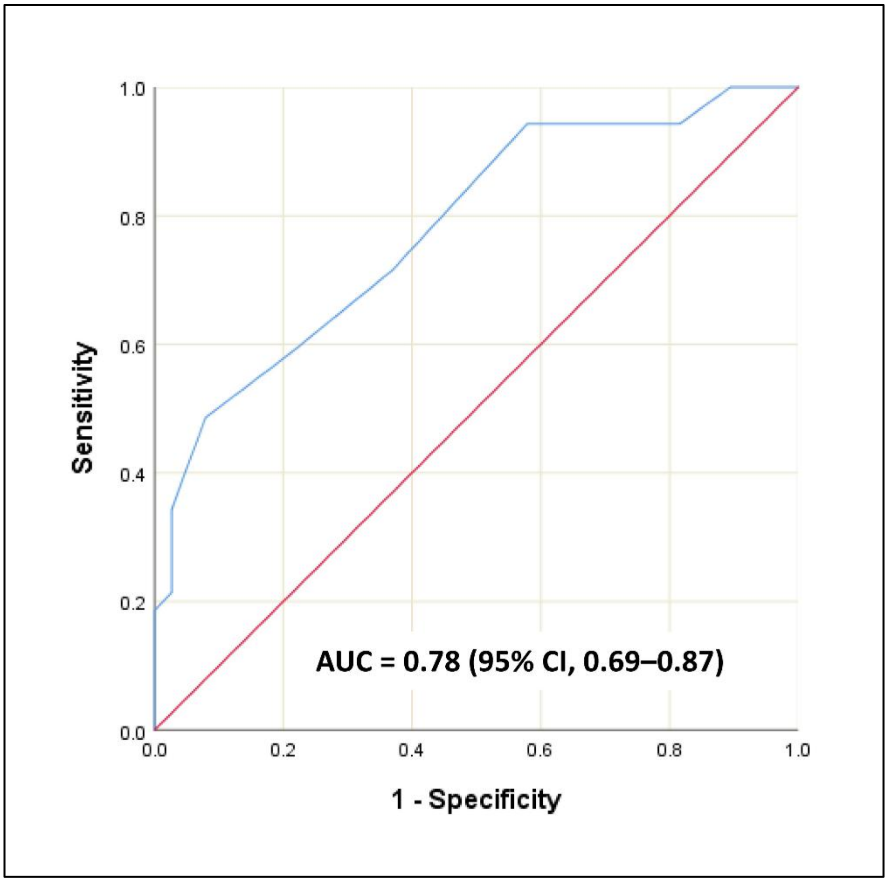

Figure S1. Receiver operating characteristic (ROC) curve for predicting upper facial weakness in patients with central facial palsy due to acute ischemic stroke. The ROC curve illustrates the performance of the multiple logistic regression model evaluating the association between selected predictors and the likelihood of upper facial weakness in patients with central facial palsy following acute ischemic stroke.
