# Supplementary material for: Analysis of Upper Facial Weakness in Central Facial Palsy Following Acute Ischemic Stroke
Source: Neurol Int. 2025 Jan 19;17(1):12. doi: 10.3390/neurolint17010012 (PMC11767383; doi:10.3390/neurolint17010012)
Supplement: Supplementary file 1 [file neurolint-17-00012-s001.zip › Table S1.pdf]

**Table S1.** Multicollinearity analysis of candidate variables for multiple logistic regression*

| Variable | Collinearity Statistics | |
|---|---|---|
| | **Tolerance** | **Variance inflation factor (VIF)** |
| NIHSS score | 0.738 | 1.355 |
| Lower facial weakness | 0.804 | 1.244 |
| Lateral middle cerebral artery cortex (M5) lesion† | 0.801 | 1.248 |
| Lentiform nucleus lesion† | 0.799 | 1.251 |
| Anterior cerebral artery circulation lesion† | 0.905 | 1.105 |
| Cerebellar lesion† | 0.844 | 1.185 |
| Lesion side | 0.904 | 1.106 |
| Dilated cardiomyopathy | 0.877 | 1.140 |

* Multiple logistic regression analysis was performed to identify the factors associated with the upper facial weakness in patients with central facial palsy due to acute ischemic stroke. Collinearity was assessed using tolerance and variance inflation factor (VIF) statistics. NIHSS denotes National Institutes of Health Stroke Scale.

† Lesion location was determined by the Alberta Stroke Program Early Computed Tomography Score (ASPECTS) system encompassing the anterior cerebral artery circulation and the posterior circulation Acute Stroke Prognosis Early CT score (pc-ASPECTS) system.
